# All About Clonidine and Its Role in Preventing Hematoma Following Aesthetic Facial Surgery: A Narrative Review With Practical Guidance

**DOI:** 10.1093/asjof/ojag130

**Published:** 2026-06-24

**Authors:** Dillon C Mobasser, Alex N Kerendi, Foad Nahai

## Abstract

Postoperative hematoma remains the most common major complication following face and necklift surgery, with decades of literature highlighting perioperative hypertension and sympathetic surges as primary modifiable risk factors. Clonidine, a centrally acting alpha-2 adrenergic agonist, has been progressively incorporated into facelift protocols to stabilize postoperative blood pressure and blunt sympathetic activation. This narrative review examines the pharmacology of clonidine, the pathophysiology of postfacelift bleeding, and the clinical evidence supporting clonidine's role in perioperative blood pressure management. Practical dosing strategies, contraindications, and safety considerations are presented based on the available literature and the authors’ clinical experience. When used in a comprehensive perioperative blood pressure management protocol, clonidine appears to be a safe and effective adjunct for hematoma prevention in aesthetic surgery, although the supporting evidence is predominantly Level IV (retrospective series and observational studies) supplemented by expert opinion. Practical dosing strategies, contraindications, and safety considerations are presented based on the available literature and the authors’ clinical experience.

Level of Evidence: 5 (Therapeutic)

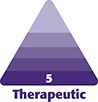

Hematoma has been widely recognized as the most frequent and clinically consequential complication following face and necklift surgery, with rates ranging from 3% to 14%.^1,2^ Early studies showed hematoma to be a defining challenge in facial rejuvenation surgery. For example, Straith et al reported on 13 hematomas in 500 consecutive facelift surgeries, firmly establishing postoperative bleeding as a primary complication.^3^ Later studies continued to identify hematoma as the most common major postoperative event following rhytidectomy.^4,5^ Over time, many technical strategies have been developed to mitigate hematoma risk, including closed suction drains, compression dressings, fibrin sealants, hemostatic nets, and cooling devices.^4-7^ However, despite refinements in surgical technique and management, hematoma remains a prevalent complication. Among these adjunctive measures, the hemostatic net has gained increasing acceptance, with several series reporting near-complete prevention of hematoma. Janssen et al demonstrated a reduction in hematoma rates from 3.9% to 0.6% in a 5-year single-surgeon experience of 663 patients.^8^ A recent systematic review and meta-analysis by Ribeiro et al reported a pooled hematoma incidence of 0.14 per 100 observations with hemostatic net use across 1617 patients.^9^ Although not universally adopted, the hemostatic net represents an important component of the multimodal approach to hematoma prevention and should be considered alongside pharmacologic strategies. Niamtu's comprehensive review emphasized that even in modern practice, expanding hematomas remain a clinically significant concern following facelift.^2^ Recently, the focus has shifted from solely technical factors to physiologic variables, like perioperative hypertension and sympathetic surges, as the leading drivers of postoperative bleeding. Seminal work that identified postoperative hypertension as a primary etiologic factor prompted emphasis on pharmacologic blood pressure management as a preventive strategy. Recent studies have shown that strict perioperative systolic blood pressure control, often <120 mm Hg, is associated with dramatically reduced hematoma rates.^1,10^ In this evolving framework, clonidine has become a valuable adjunct for perioperative blood pressure stabilization. This review aims to contextualize clonidine within the evolution of facelift hematoma prevention and to provide a practical guide for its use in aesthetic surgery. Although the current evidence base is centered on rhytidectomy, the physiologic rationale for perioperative blood pressure control with clonidine applies broadly across aesthetic surgery, and the senior author has incorporated this approach into all aesthetic procedures.

## PATHOPHYSIOLOGY OF POSTRHYTIDECTOMY HEMATOMA

Modern evidence consistently indicates that hematoma following face and necklift surgery is a predominantly hemodynamic complication rather than a disorder of coagulation or hemostasis. Systematic reviews confirm hematoma as the most common complication following rhytidectomy.^4,5^ Several clinical studies have identified elevated blood pressure in the postoperative period as the primary modifiable risk factor for hematoma formation.^1,10,11^ Berner et al were among the first to clearly identify postoperative hypertension as an etiologic factor in facelift hematoma, demonstrating that pharmacologic blood pressure control reduced hematoma incidence.^12^ Subsequent studies confirmed that both median and peak systolic blood pressures strongly correlate with hematoma risk.^13^ Large long-term studies, particularly in male patients, further demonstrated the association between elevated blood pressure and hematoma requiring surgical evacuation.^14^ These findings highlight the vulnerability of the facial and cervical vessels, which poorly tolerate sudden increases in arterial or venous pressures. Pain, anxiety, coughing, nausea, vomiting, bladder distention, and emergence from anesthesia can all trigger sympathetic surges capable of causing transient, but clinically significant, blood pressure spikes, leading to bleeding in undermined tissue planes.^2,15,16^ Therefore, hematoma following facelift is best conceptualized as a hemodynamic complication caused by sympathetic activation and blood pressure elevations.

## PHARMACOLOGY OF CLONIDINE: MECHANISM AND HEMODYNAMIC EFFECTS

Clonidine is a centrally acting alpha-2 adrenergic receptor agonist that dampens sympathetic outflow by decreasing the release of norepinephrine from presynaptic neurons. This central sympatholytic effect lowers heart rate, blood pressure, and peripheral vascular resistance.^17-20^ In addition to its central mechanism, clonidine also stimulates peripheral presynaptic alpha-2 adrenergic receptors located on sympathetic nerve terminals. Activation of these peripheral receptors inhibits norepinephrine release at the neuroeffector junction, providing a complementary peripheral sympatholytic effect that contributes to the overall reduction in sympathetic tone.^17-20^ Oral clonidine lowers blood pressure within 30 to 60 min, with peak effects around 2 to 4 h after ingestion. Heart rate reduction is typically modest with no reflex tachycardia. In addition to hemodynamic effects, clonidine also provides anxiolytic and analgesic benefits without clinically significant respiratory depression.^15^ Experimental and critical care literature also shows clonidine's ability to blunt prolonged postoperative sympathetic responses.^21^ Clonidine's pharmacologic properties make it well suited to manage the physiologic causes of postrhytidectomy hematoma by lowering blood pressure and reducing sympathetic surges. Consistent with these properties, a systematic review and meta-analysis by Sanchez Munoz and colleagues confirmed that perioperative clonidine improves hemodynamic stability, reduces analgesic consumption by 24%, and decreases postoperative nausea and vomiting, with no adverse effects on renal function or awakening time.^22^

## DURATION OF ACTION: ORAL VS TRANSDERMAL FORMS

Oral clonidine's duration of action is ∼6 to 12 h, requiring repeated dosing for sustained blood pressure control.^17-20,23^ In contrast, transdermal clonidine delivers medication continuously over several days, reaching steady therapeutic plasma concentrations after ∼48 to 72 h.^17^ Clinical facelift protocols commonly apply the patch preoperatively and continue use for 72 h after surgery to cover the period of highest hematoma risk.^10^ This sustained delivery system is advantageous for maintaining stable postoperative blood pressure and minimizing sympathetic surges during the susceptible early postoperative period. A summary of oral vs transdermal clonidine is summarized in Table 1.

**Table 1.** Oral vs Transdermal Clonidine

	Oral clonidine	Transdermal clonidine
Dose	0.1-0.2 mg	0.1-0.3 mg/day
Onset	30-60 min	12-24 h
Peak effect	2-4 h	Steady state at 2-3 days
Duration	6-12 h	Up to 7 days
Half-life	12-16 h (elimination)	∼20 h after patch removal
Use	Postoperative BP spikes	Perioperative BP stabilization
Advantages	Rapid BP control	Continuous delivery
Risks and considerations	Hypotension, bradycardia; avoid abrupt discontinuation in chronic users	Hypotension, bradycardia, delayed onset; apply preoperatively

Data derived from Ramanadham et al,^10^ DailyMed,^17-20^ and Beninger and Pritchard.^23^ BP, blood pressure.

## MECHANISMS OF HEMATOMA REDUCTION

**Figure 1.**
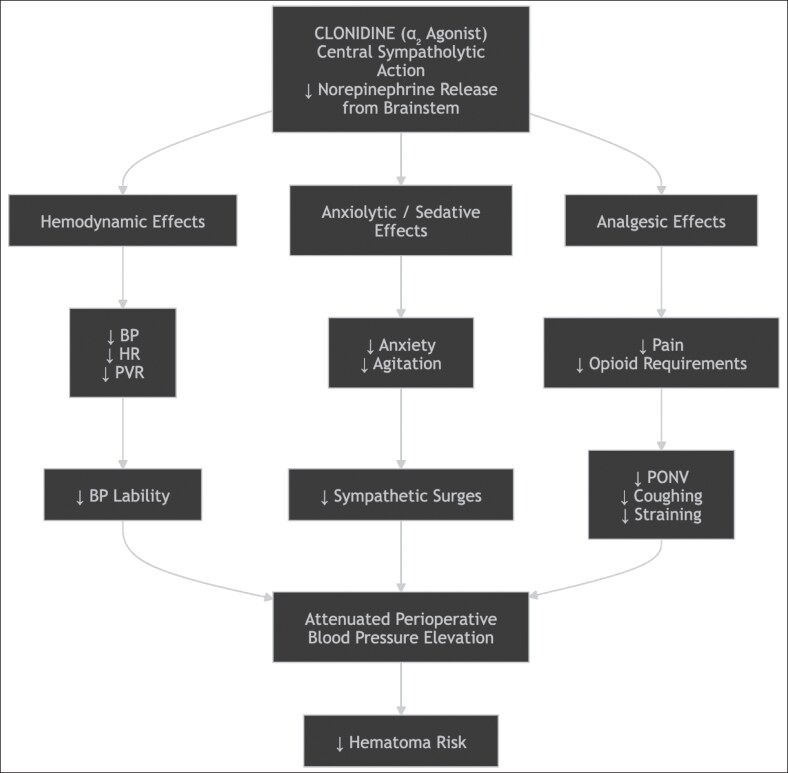
Mechanism of clonidine in reducing postrhytidectomy hematoma. Clonidine acts centrally (and to a lesser extent peripherally) to reduce sympathetic outflow, producing complementary hemodynamic, anxiolytic, and analgesic effects that converge to attenuate perioperative blood pressure elevation. BP, blood pressure; HR, heart rate; PONV, postoperative nausea and vomiting; PVR, peripheral vascular resistance.

Clonidine reduces hematoma risk through multiple complementary mechanisms. Central sympatholysis leads to sustained stabilization of perioperative blood pressure and reduction of catecholamine surges.^23^ Its anxiolytic and analgesic properties further reduce postoperative pain, agitation, coughing, and nausea, which are known triggers of acute blood pressure elevation.^15,16^ Multimodal prophylactic protocols utilizing clonidine have demonstrated significant reductions in acute postoperative hematoma, supporting the importance of hemodynamic stabilization as the underlying mechanism.^15,16^ These properties make clonidine an effective adjunct medication by addressing the physiological causes of postfacelift bleeding (Figure 1).

## CLINICAL EVIDENCE IN FACE AND NECKLIFT SURGERY

There is a growing body of clinical evidence, predominantly from retrospective case series and observational studies (Levels III and IV), supporting clonidine use as part of multimodal perioperative protocols in rhytidectomy. Beninger and Pritchard showed improved blood pressure control and reduced hematoma rates in patients receiving clonidine during facelift surgery.^23^ Ramanadham et al reported a large series of 1089 facelift patients managed with a predetermined antihypertensive protocol incorporating clonidine, resulting in hematoma rates <1%.^10^ Beer and colleagues demonstrated that multimodal prophylactic regimens including clonidine lowered acute postoperative hematoma rates from 7% to 0%.^16^ More recently, Bassiri-Tehrani et al showed that strict control of systolic blood pressure <120 mm Hg dramatically reduced hematoma rates.^1^ Additional contemporary protocols using multimodal strategies for blood pressure control achieved hematoma rates <0.3%.^6,24^ It should be noted that in all cited studies, clonidine was used as one component of multimodal perioperative protocols. The observed reductions in hematoma rates cannot be attributed to clonidine alone but rather to the overall strategy of strict blood pressure control, of which clonidine is a key pharmacologic facilitator.

## PRACTICAL USE AND DOSING STRATEGIES

Clonidine can be administered orally, transdermally, or in combination. For acute postoperative blood pressure elevations, oral dosing of 0.1 to 0.2 mg is commonly used for rapid reduction.^18-20,23^ Transdermal patches that deliver 0.1 to 0.3 mg per day provide sustained control and are frequently applied preoperatively and continued 72 h after surgery.^10,17^ When transdermal clonidine is used, the patch should be applied ∼24 h preoperatively to allow adequate time for drug absorption, given the 12 to 24 h delay before therapeutic plasma levels are achieved.^17^ In the context of perioperative use, “long-term use” generally refers to continuous administration for ∼1 week or longer, at which point abrupt discontinuation may carry a risk of rebound hypertension. For patients who have received clonidine for 1 week or more, a gradual taper is recommended, reducing the dose by 0.1 mg every 1 to 2 days, rather than abrupt cessation, consistent with FDA labeling.^17-20^ Many surgeons use a threshold-based approach, administering oral clonidine when systolic blood pressure exceeds 120 to 140 mm Hg postoperatively. This aligns with modern evidence emphasizing the importance of strict blood pressure control.^1^ Dosing strategies and key clinical considerations for perioperative clonidine use in aesthetic surgery are summarized in Table 2.

**Table 2.** Practical Use and Dosing Strategies

Category	Recommendation	Rationale	References
Indications	HTN; BP Lability; SBP >120-140 mm Hg	Elevated BP is a primary modifiable risk factor	^1, 10, 11, 13, 14^
Oral dosing	0.1-0.2 mg PO PRN	Rapid BP control	^10, 18-20, 23^
Transdermal dosing	0.1-0.3 mg/day patch for ∼72 h	Sustained sympatholysis	^1, 10, 11, 17^
Heart rate effects	Mild HR reduction; monitor if coadministration with β-blockers	Avoid bradycardia	^10, 17-20^
Contraindications	Hypotension; AV block; hypersensitivity	Safety	^17-20^
Rebound prevention	Avoid abrupt discontinuation in long-term use	Prevent rebound hypertension	^17-20, 25, 26^

Recommendations represent the authors’ synthesis of available literature and expert clinical experience. AV, atrioventricular; BP, blood pressure; HR, heart rate; HTN, hypertension; PO, by mouth; PRN, as needed; SBP, systolic blood pressure.

## SAFETY, DRUG INTERACTIONS, AND CONTRAINDICATIONS

Clonidine is generally well tolerated. Absolute contraindications include hypersensitivity, severe hypotension, and advanced atrioventricular block. Caution is advised when used in patients with sinus node dysfunction and those receiving beta blockade, because of the potential for bradycardia and atrioventricular conduction abnormalities.^17-20^ Drug interactions include additive hypotensive effects with other antihypertensive agents, increased sedation with opioids and sedatives, and potential reduction in effect when administered with tricyclic antidepressants.^17-20^ When clonidine is used perioperatively, blood pressure and heart rate should be assessed before each dose and at regular intervals during the postoperative period. Oral clonidine should be withheld if systolic blood pressure falls <90 mm Hg or heart rate drops <60 beats per minute, and the transdermal patch should be removed if sustained hypotension or symptomatic bradycardia develops. Sedation level should also be monitored when clonidine is combined with central nervous system depressants, as additive effects may occur.^17-20,22^

## REBOUND HYPERTENSION AND GUIDELINE CONTEXT

Abrupt discontinuation of clonidine may lead to rebound hypertension because of a surge in sympathetic activity. This phenomenon is most relevant in patients receiving chronic clonidine therapy and is rare with short-term perioperative use. Current cardiovascular guidelines advise against abrupt clonidine withdrawal and emphasize continuation through the perioperative period when clinically indicated.^17-20,25,26^ Importantly, guidelines that recommend against routine clonidine initiation for cardiovascular risk reduction in noncardiac surgery do not address its targeted use for blood pressure control to prevent hematoma in aesthetic surgery.^25^

## INTEGRATION INTO FACELIFT PROTOCOLS

**Figure 2.**
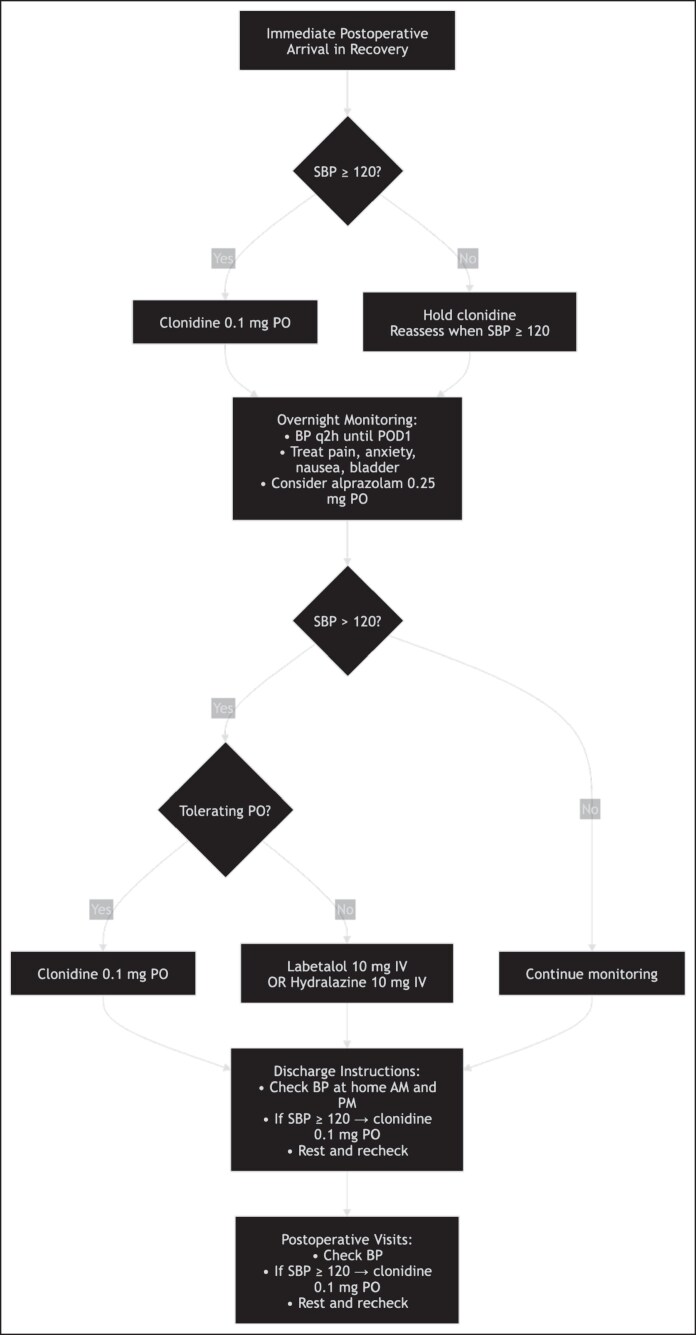
Proposed perioperative blood pressure management protocol incorporating clonidine for face and necklift surgery. This protocol represents the authors’ recommended approach based on available evidence and clinical experience. Adapted from Bassiri-Tehrani et al.^1^ BP, blood pressure; PO, by mouth; IV, intravenous; POD, postoperative day; Rx, prescription; SBP, systolic blood pressure.

Clonidine is most effective when integrated into multimodal hematoma prevention protocols. Modern hematoma prevention is multifactorial, encompassing strict perioperative blood pressure control, adequate analgesia, antiemetic therapy, sedation, meticulous surgical hemostasis, and adjunctive measures, such as fibrin sealants, hemostatic nets, and tranexamic acid.^5-7,10,16,24,27^ Within this framework, clonidine functions as a pharmacologic adjunct that addresses the hemodynamic component of hematoma risk, rather than a standalone solution (Figure 2).

## CLONIDINE'S ROLE IN PATIENTS ON CHRONIC ANTIHYPERTENSIVE THERAPY

Patients who are already receiving antihypertensive medication require careful perioperative planning. Current cardiovascular guidelines emphasize the importance of continuing chronic blood pressure therapy throughout the perioperative period to avoid blood pressure instability and withdrawal phenomena.^25,26^ This principle is particularly relevant for clonidine, as abrupt discontinuation in long-term users may precipitate rebound hypertension because of a surge in sympathetic activity.^25,26^

In patients already taking clonidine, continuation of therapy through the perioperative period is strongly recommended. If oral intake is interrupted, switching to a transdermal formulation is a practical and effective way to maintain stable plasma drug levels and prevent withdrawal-related hypertension.^17-20^

In patients receiving other classes of antihypertensive agents, clonidine may be safely added as an adjunct for perioperative blood pressure control, as its mechanism acts centrally rather than peripherally. However, careful monitoring is required when combined with β-blockers or other agents that affect atrioventricular nodal conduction, because of the potential for bradycardia and other conduction abnormalities.^17-20^

Clonidine serves as a rapid, predictable, and titratable option for treating postoperative hypertension in both normotensive and chronically hypertensive patients, making it a valuable option for modern facelift protocols that target strict systolic blood pressure thresholds.^1,10,11^

## CLONIDINE'S ROLE IN THE SENIOR AUTHOR'S PRACTICE

The following section reflects Level V evidence (expert opinion) based on the senior author's clinical experience. In the senior author's current practice, clonidine has become a central component of perioperative blood pressure management for all aesthetic surgery. It is routinely incorporated into the standard hematoma prevention protocol for all patients, with the goal of maintaining strict systolic blood pressure control throughout the perioperative and early postoperative periods.

This protocol-based approach reflects the growing body of evidence that demonstrates that meticulous blood pressure control is among the most important modifiable factors in reducing hematoma risk.^1,6,10,11^ Bassiri-Tehrani et al reported no clonidine-related complications in a large contemporary series using strict systolic blood pressure targets, which is consistent with the senior author's experience, where no clinically significant adverse events attributable to clonidine have been observed.^1^

In the senior author's experience, beyond what has been reported in the published series, clonidine provides reliable perioperative blood pressure stabilization, reduced need for rescue antihypertensive medications, smoother emergence and recovery, and improved patient comfort.^1^ Importantly, its use has been associated with sustained reduction in postoperative hematoma rates without an increase in the incidence of bradycardia, hypotension requiring intervention, or delayed recovery period. These observations support clonidine as a safe and consistent component of the modern, protocol-driven approach to hematoma prevention in aesthetic surgery.

## OTHER APPLICATIONS OF CLONIDINE

Beyond its role for hemodynamic control in aesthetic surgery, clonidine has a wide range of FDA-approved and off-label clinical applications that further underscore its safety and versatility. Clonidine is approved by the FDA for the treatment of elevated blood pressure and, in its extended-release formulation, for attention-deficit hyperactivity disorder (ADHD).^17-20,28^

Extended-release clonidine is approved for ADHD management as monotherapy or as an adjunct to stimulant medications. Although the exact mechanism in this context remains incompletely understood, it is believed that the clinical benefit is partially associated with the modulation of noradrenergic activity, potentially involving prefrontal cortical effects rather than direct stimulant effects.^18-20^ These central sympatholytic and sedative properties align with the anxiolytic and emergence-modulating effects that make clonidine appealing for the perioperative facelift population.

Among its established off-label indications is the management of opioid withdrawal. Alpha-2 agonists reduce autonomic hyperactivity, including hypertension and tachycardia, and improve symptoms such as anxiety and myalgias. Although it remains a second-line option, as it does not relieve opioid withdrawal symptoms as effectively as buprenorphine and methadone, it provides significant symptomatic relief compared with placebo when first-line agents are not available.^29^ Clonidine has also shown efficacy in reducing tic severity in patients with Tourette syndrome, especially those with comorbid ADHD, although sedation and dose-dependent hypotension may limit its use in some populations.^30,31^

Clonidine has also been utilized for the management of menopausal vasomotor symptoms, resulting in modest reductions in hot flash frequency at low daily doses without clinically significant hypotension.^32^ In the field of addiction medicine, clonidine has shown benefit as a non-nicotine pharmacologic aid for smoking cessation. It has achieved higher short-term abstinence rates in patients with heavy tobacco dependence who had not responded to standard therapies.^33^ Additional applications include the management of posttraumatic stress disorder-related hyperarousal, neuroleptic-induced akathisia, stimulant-associated insomnia, and clozapine-induced sialorrhea, although the strength of evidence in these settings is variable.^28^

The breadth of these indications demonstrates a consistent pharmacologic theme: reduction in central sympathetic tone and modulation of noradrenergic activity. This long history of use across multiple medical specialties, such as psychiatry, addiction medicine, neurology, and cardiovascular care, provides substantial real-world safety experience, supporting the perioperative use of clonidine in appropriately selected individuals for aesthetic surgery.

## LIMITATIONS

A systematic review was not undertaken because the existing literature on clonidine in rhytidectomy is embedded within multimodal perioperative protocols, and clonidine's independent contribution cannot be reliably isolated from other concurrent interventions. The heterogeneity of study designs, protocols, and outcome reporting would preclude meaningful quantitative synthesis.

The existing literature supporting clonidine use for hematoma prevention in facelift surgery is predominantly composed of retrospective case series and observational studies (Level IV) supplemented by expert opinion (Level V) on the Oxford Centre for Evidence-Based Medicine hierarchy. There are no randomized controlled trials specifically evaluating clonidine for hematoma prevention in rhytidectomy. Additionally, the clinical studies cited employ multimodal protocols in which clonidine is one of several interventions, making it difficult to isolate its independent contribution to hematoma reduction. The observed reductions in hematoma rates in the cited studies cannot be attributed to clonidine alone but rather to the overall strategy of strict blood pressure control, of which clonidine is a key pharmacologic facilitator. The largest randomized controlled trial of perioperative clonidine, as discussed in current perioperative cardiovascular guidelines, evaluated cardiovascular risk reduction in major noncardiac surgery rather than blood pressure control for surgical bleeding prevention, and its findings are not directly applicable to the aesthetic surgery population.^25^ Despite the absence of higher-level evidence, the physiologic rationale for clonidine use is well established: postoperative hypertension is the dominant modifiable risk factor for hematoma, and clonidine directly targets this mechanism through central sympatholysis. The consistent clinical experience across multiple large series demonstrating hematoma rates <1% when strict blood pressure protocols incorporating clonidine are employed provides compelling support for its efficacy.^1,10,16,22,24^

## CONCLUSIONS

Clonidine directly targets the physiological drivers of postoperative hematoma through central sympatholysis, blood pressure stabilization, and attenuation of perioperative catecholamine surges. A growing body of clinical experience supports its role in modern facelift hematoma prevention protocols. When used sensibly and integrated into comprehensive perioperative management strategies, clonidine appears to be a safe, effective pharmacological adjunct for hematoma prevention in aesthetic surgery.
